# Nestin-GFP Transgene Reveals Neural Precursor Cells in Adult Skeletal Muscle

**DOI:** 10.1371/journal.pone.0016816

**Published:** 2011-02-03

**Authors:** Alexander Birbrair, Zhong-Min Wang, Maria Laura Messi, Grigori N. Enikolopov, Osvaldo Delbono

**Affiliations:** 1 Department of Internal Medicine-Gerontology, Wake Forest University School of Medicine, Winston-Salem, North Carolina, United States of America; 2 Neuroscience Program, Wake Forest University School of Medicine, Winston-Salem, North Carolina, United States of America; 3 Cold Spring Harbor Laboratory, Cold Spring Harbor, New York, United States of America; Brigham & Women's Hospital, United States of America

## Abstract

**Background:**

Therapy for neural lesions or degenerative diseases relies mainly on finding transplantable active precursor cells. Identifying them in peripheral tissues accessible for biopsy, outside the central nervous system, would circumvent the serious immunological and ethical concerns impeding cell therapy.

**Methodology/Principal Findings:**

In this study, we isolated neural progenitor cells in cultured adult skeletal muscle from transgenic mice in which nestin regulatory elements control GFP expression. These cells also expressed the early neural marker Tuj1 and light and heavy neurofilament but not S100β, indicating that they express typical neural but not Schwann cell markers. GFP+/Tuj1+ cells were also negative for the endothelial and pericyte markers CD31 and α-smooth muscle actin, respectively. We established their a) functional response to glutamate in patch-clamp recordings; b) interstitial mesenchymal origin; c) replicative capacity; and d) the environment necessary for their survival after fluorescence-activated cell sorting.

**Conclusions/Significance:**

We propose that the decline in nestin-GFP expression in muscle progenitor cells and its persistence in neural precursor cells in muscle cultures provide an invaluable tool for isolating a population of predifferentiated neural cells with therapeutic potential.

## Introduction

Hopes for finding a therapy to replace lost or defective neuronal and/or glial cells, thus healing neural lesions or degenerative diseases, rest mainly on stem cells as transplantable active precursors. Primarily derived from embryonic and fetal tissues, their clinical use is limited by ethical and technical concerns related to heterologous transplants [1], [2]. Autologous tissues would be a valid alternative, but neural precursors from the central or peripheral nervous system cannot be obtained without potentially harmful consequences for the donor.

Interest in adult human stem cells arises from recent work demonstrating their ability to differentiate into various lineages [3], [4], [5] [6]. Multipotent cells, reminiscent of embryonic neural stem cells, have been isolated from several postnatal tissues, including skeletal muscle, skin, gut, heart, fat, and dental pulp, with promise for applications in research and therapy [7], [8], [9], [10], [11] [12], [13]. Several markers are used to identify neuronal precursor cells in the nervous system; one is nestin [14], an intermediate protein classified as a type IV neurofilament, which, together with microfilaments and microtubules, constitutes most of the cytoskeleton. Previous work has shown that the nestin-GFP transgene is a useful marker of neurogenesis throughout life [15], and GFP fluorescence can be used to trace the migration and differentiation of newly generated neurons in the mammalian central nervous system [16].

The persistence of neural stem cells in skeletal muscle raises the possibility of their use for a variety of research purposes and therapies. To identify and isolate a transplantable population highly enriched in neural precursor cells, we used a transgenic mouse in which nestin regulatory elements control GFP expression [15]. Here, we show that nestin is co-expressed with other neuronal stem cell markers in the skeletal muscle and that its presence can facilitate their identification and isolation from adult skeletal muscle or neurosphere cultures by fluorescence-activated cell sorting (FACS).

Previous studies using immunostaining, RNA extraction, western blot, and antibody-based FACS revealed that stem cells residing in adult muscle differentiate into neurons [7], [11], [17]. However, these procedures prevent their use for culture, cloning, or transplant. More important, functional identity, unequivocal source, replicative capacity, and survival after FACS are unknown. The decline in nestin-GFP expression in muscle progenitor cells coupled to its persistence in neural precursor cells indicates the discovery of a new tool to address these clinically relevant problems.

## Materials and Methods

### Animals

Nestin-GFP transgenic mice were maintained as homozygous for the transgene on the C57BL/6 genetic background [15]. C57BL/6 wild-type mice were used as the control. All mouse colonies were housed at Wake Forest University School of Medicine (WFUSM) in a pathogen-free facility of the Animal Research Program under 12∶12-h light/dark cycle and fed ad libitum. Both male and female homozygous mice were used, and their ages ranged from 3 to 5 months. Mice were sacrificed by cervical dislocation after isoflurane anesthesia.

### Ethics Statement

Animal handling and procedures were approved by WFUSM Animal Care and Use Committee protocol A10-159.

### Cell cultures

A pool of hindlimb muscles; flexor digitorum brevis (FDB) muscle only; extensor digitorum longus (EDL) muscle satellite cell culture; and primary Schwann cells were used as indicated for each experiment. The pooled hindlimb muscles were used when a large number of mesenchymal cells was needed. The FDB muscle was preferred over more traditional muscles for most experiments because it is small and flat, allowing more complete dissociation by trituration in a single step, and shortening the experiment significantly. EDL myofibers were used to isolate exclusively nestin-GFP+ satellite cells. The primary Schwann cell culture was used as a positive control for the S100β antibody. All muscle-derived cultures were grown on laminin-coated coverslips in tissue culture plates.

#### Pooled hindlimb muscle preparation

The preparation includes all hindlimb muscles except for those in the foot. It was used for FACS on the day of muscle dissociation or at later times, followed by cell culture, as explained below. Hindlimb muscles were carefully dissected away from the surrounding connective tissue and minced. They were digested by gentle agitation in 0.2% (w/v) type-2 collagenase in Krebs solution at 37°C for 2 hours, then dissociated by trituration and resuspension in 0.25% trypsin/0.05% EDTA in PBS for 15 minutes at 37°C. After centrifuging at1500 rpm for 5 minutes, the supernatant was removed, and the pellet resuspended in *resuspension medium* consisting of Dulbecco's modified Eagle's medium (DMEM), containing 2 mM L-glutamine and 1% penicillin/streptomycin, supplemented with 10% (v/v) horse serum (Invitrogen, Eugene, OR) and 0.5% (v/v) chicken embryo extract (CEE, Gemini Bio-products, West Sacramento, CA). Suspended cells were passed through a 40 µm cell strainer (BD Biosciences, Mississauga, Ontario, Canada) and used for FACS and culture.

For FACS experiments performed 7–14 days after muscle dissociation, cultured nestin-GFP+ cells were washed with phosphate-buffered saline (PBS) and treated with 0.25% trypsin/0.05% EDTA to isolate them in a suspension. When all cells were detached, the enzymatic reaction was stopped with the *resuspension medium* described above. We applied mechanical trituration using fire-polished glass pipettes to increase cell dissociation. Cells were centrifuged at 1000 rpm for 5 min, and the pellet was resuspended in DMEM at 10^6^ cells/ml. Aggregates were removed by passing them through a 40 µm cell strainer prior to sorting.

#### Flexor digitorum brevis (FDB) preparation

FDB muscle from nestin-GFP transgenic and wild-type mice was used for most experiments in this work. Muscles were carefully dissected away from the surrounding connective tissue and digested by gentle agitation in 0.2% (w/v) Worthington's type-2 collagenase in Krebs solution at 37°C for 2 hours. They were resuspended in *resuspension medium* and dissociated by gentle trituration. The growth medium used to plate cell cultures consisted of DMEM-high glucose (Invitrogen), supplemented with 2% L-glutamine, 50 U/ml penicillin and 50 mg/ml streptomycin, and 10% (v/v) horse serum (Invitrogen) and 0.5% (v/v) CEE (Gemini Bio-products) [18]. It supported both proliferation and differentiation of myogenic cells [18].

#### Extensor digitorum longus (EDL) muscle satellite cell culture

EDL muscles were carefully dissected from tendon to tendon; connective tissue and fat were removed, and collagenase digested, as described above. Single myofibers were dissociated by gentle trituration with a fire-polished Pasteur pipette. Single, intact myofibers exhibiting associated GFP+ satellite cells and with no other fluorescence cells/debris attached were transferred through a series of four 60 mm Petri dishes filled with DMEM to slough loose interstitial GFP+ cells. Myofibers (50–80) were treated with trypsin/EDTA 0,125% for 15 minutes at 37°C and filtered through a 40 µm cell strainer. Filtered cells were cultured in F10 Ham medium (Invitrogen) supplemented with 20% FBS, 2 mM L-glutamine, 50 U/ml penicillin and 50 mg/ml streptomycin (Invitrogen), and 5 ng/ml basic Fibroblast Growth Factor (Promega, Madison, WI).

#### Primary Schwann cell culture

Primary mouse Schwann cells, isolated from early postnatal (P2-P8) C57BL/6 mouse sciatic nerves, were purchased from ScienCell Research Laboratories and cultured in Schwann cell medium (ScienCell Research Lab, Carlsbad, CA). They were plated on 35-mm dishes coated with 50 µg/ml poly-L-lysine following the company's protocol.

### Immunocytochemistry, skeletal muscle histology, and cell-proliferation assay

For immunostaining, culture cells were fixed with 4% paraformaldehyde (PFA) for 30 minutes, then permeabilized in 0.5% Triton X-100 (Sigma, St. Louis, MO), and blocked to saturate nonspecific antigen sites using 5% (v/v) goat serum/PBS (Jackson Immunoresearch Labs, West Grove, PA) overnight at 4°C. The next day, the cells were incubated with primary antibodies at room temperature for 4 h and visualized using appropriate species-specific secondary antibodies conjugated with Alexa Fluor 488, 568, or 647 at 1∶1000 dilution (Invitrogen). They were counterstained with Hoechst 33342 reagent at 1∶2000 dilution (Invitrogen) to label the DNA and mounted on slides for fluorescent microscopy with Fluorescent Mounting Medium (DakoCytomation, Carpinteria, CA).

For histological analysis of nestin-GFP and Pax7 expression in the nestin-GFP transgenic mouse, we used EDL muscle, whose fibers, in contrast to FDB, exhibit a simple pennation. The complexity of FDB fiber pennation precludes sharp, uniform perpendicular cross-sections. EDL muscles from nestin-GFP transgenic mice were dissected, prefixed in 2% PFA in 0.1 M of PBS at pH 7.4 for 24 h at 4°C, and cryoprotected with sucrose, as described [19]. Muscles were permeabilized with 0.5% Triton X-100 in PBS, blocked with 10% goat serum at 4°C overnight, exposed to Pax7 antibody (1/100 dilution) plus 1% goat serum for 3 h and then to the goat anti-mouse Alexa Fluor 568 (1/1000) secondary antibody. Hoechst 33342 (1/2000) and Fluorescent Mounting Medium were used as described above for cultured cells.

For cell proliferation, we used an EdU (5-ethynyl-2′-deoxyuridine) assay kit (Invitrogen), following the manufacturer's instructions.

### Primary antibodies

The following primary antibodies were used: rabbit monoclonal anti-Tuj1 (Covance, Princeton, NJ), mouse monoclonal anti-MyoD (BD Pharmingen, San Diego, CA), mouse monoclonal anti-desmin (Sigma), rabbit polyclonal anti-S100β (GeneTex, Irvine, CA), rabbit polyclonal anti-neurofilament L (anti-NF-L; Chemicon-Millipore, Temecula, CA), rabbit polyclonal anti-neurofilament-H (anti-NF-H; Chemicon-Millipore), mouse monoclonal anti-GFP (Invitrogen), rat monoclonal anti-mouse CD31 (BD Pharmingen), mouse monoclonal anti- α smooth-muscle actin (Sigma), and mouse monoclonal anti-Pax7 (Developmental Studies Hybridoma Bank, University of Iowa, Iowa City, IA)

### Microscopy, Cell Imaging, and Counting

An inverted motorized fluorescent microscope (Olympus, IX81, Tokyo, Japan) with an Orca-R2 Hamamatsu CCD camera (Hamamatsu, Japan) was used to acquire images. Camera drive and acquisition were controlled by a MetaMorph Imaging System (Olympus). Ten arbitrary microscopic fields were counted in each immunostained plate, and values pooled from parallel duplicates per time point and individual experiment.

### Time-lapse recording

FDB muscles were digested by gentle agitation in 0.2% (w/v) type-2 Sigma collagenase diluted in Krebs solution at 37°C for 2 hours. They were then dissociated into single myofibers by trituration with a Pasteur pipette, pretreated with serum to prevent fibers from attaching to the walls. After brief centrifugation, the supernatant was removed, and the pellet resuspended in plating medium (see above). The cell suspension was cultured in flasks coated with laminin, using plating medium in 5% CO_2_ at 37°C, and followed for 4–5 days in a time-lapse system until the fluorescence disappeared.

The FDB myofibers were cultured on the stage of a time-lapse microscopy system (37°C, 5% CO_2_) using an Olympus inverted phase-contrast microscope fitted with a video camera. Cells were observed for 4–5 days, and their morphology monitored with phase-contrast. Cell fluorescence was monitored at 2-hour intervals. Pictures were recorded on standard VHS tape and digitized for analysis.

### Fluorescence-activated cell sorting

FACS was carried out on a BD FACS (Aria Sorter, San Jose, CA) at 4°C and a pressure of 20 psi, using a laser at the 488 nm line, a 530/30 band pass filter, a 100 µm sorting tip, and a 34.2 kHz drive frequency, sterilized with 10% bleach. This instrument allowed us to characterize cells by size as well as fluorescence. Low flow rate improved the purity of cell sorting. Data acquisition and analyses were performed using BD FACS Diva 5.0.3 software, gated for a high level of GFP expression. The clear separation of GFP+ from GFP- cells explains the ease of sorting. Sorted cells were re-analyzed to confirm that all were GFP+. They were then plated on laminin-coated dishes.

### Patch-clamp recording

Cells were voltage-clamped using an Axopatch-200B amplifier (Molecular Devices, Silicon Valley, CA) in the whole-cell configuration of the patch-clamp technique [20]. Nestin-GFP+ cells were cultured for 12–16 days on glass coverslips and continuously perfused with an external solution (see below), using a push-pull syringe pump (WPI). Only those exhibiting 3 or more multipolar processes were transferred to a small, flow-through Lucite chamber positioned on an Axiovert 100 (Zeiss) microscope stage. Patch pipettes were pulled from borosilicate glass (Boralex, WPI, Sarasota, FL) using a Flaming Brown micropipette puller (P97, Sutter Instrument Co., Novato, CA), then fire-polished to obtain electrode resistances ranging from 1.5 to 3.0 GΩ. The pipette was filled with the following solution (mM): 140 CsCl, 4 NaCl, 0.5 CaCl_2_, 5 K-EGTA (ethylene glycol tetraacetic acid), and 10 K-HEPES (N-[2-hydroxyethyl]piperazine-N'-[2-ethanesulfonic acid]). The composition of the bath solution was (mM): 140 NaCl, 2.8 KCl, 1.0 CaCl_2_, and 10 HEPES [21]. To test Ca^2+^ permeation through glutamate receptors, 0.5 mM MgCl_2_ was added in some experiments. Solution pH was adjusted to 7.2 with NaOH. All experiments were conducted at room temperature (21–22°C). Glutamate was flushed a few microns away from the patch-clamped cells using a Picospritzer II (General Valve Co., Pine Brook, NJ) to control the pressure and duration of the perfusion pulse.

### Statistical Analysis

Results are expressed as the mean ± SEM. Statistical significance was assessed using analysis of variance (ANOVA) followed by t-test using Prism GraphPad. *P*<0.05 was considered significant.

## Results

### Skeletal muscle-derived neural stem cells are nestin-GFP+ and Tuj1+

**Figure 1:**
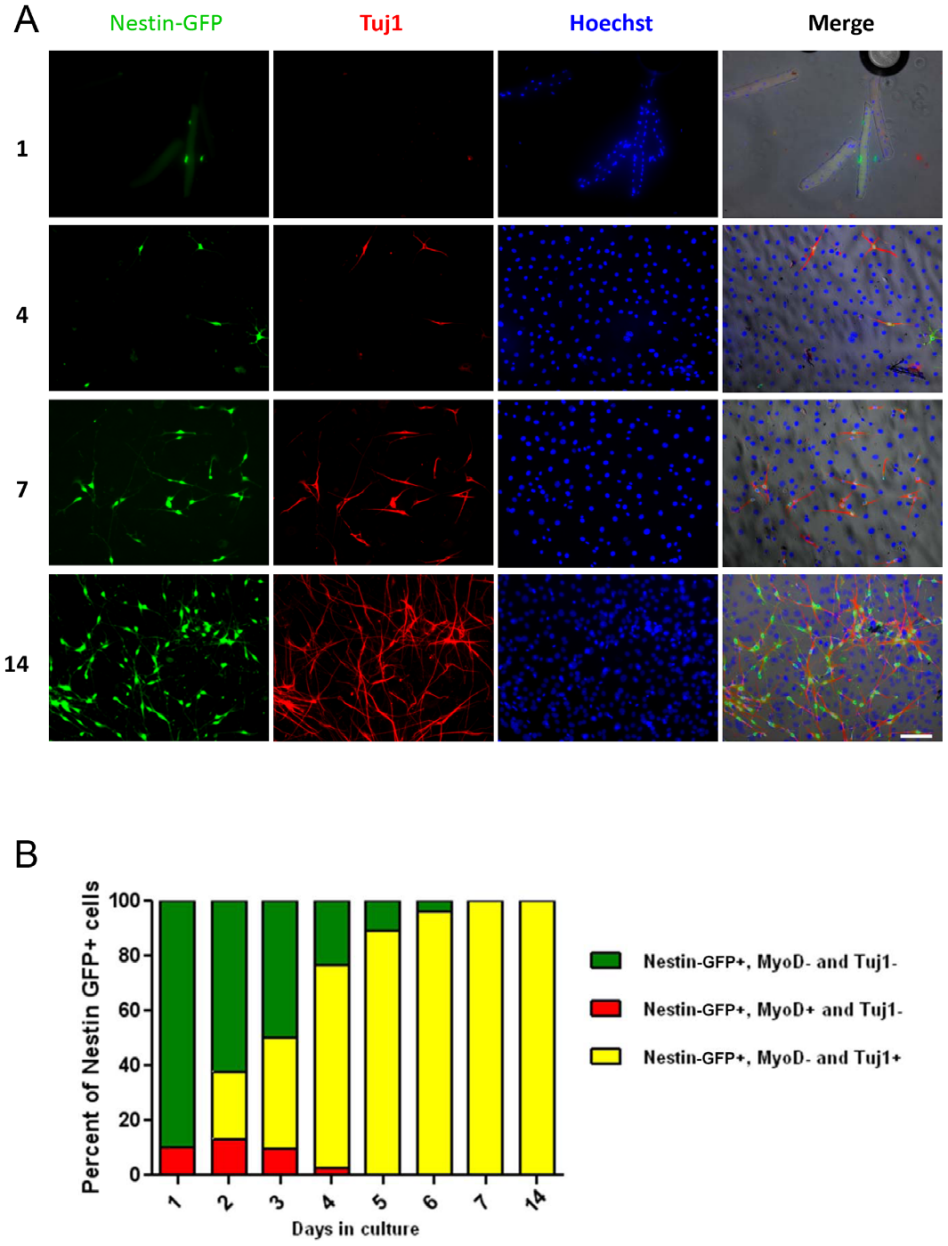
Nestin-GFP+ cells from FDB muscle in culture. **A.** Cells were grown for 14 days and fixed at various times (1, 4, 7 and 14 days). Nestin-GFP, Tuj1, and Hoechst 33342 were merged with phase contrast. Scale bar  = 100 µm. **B.** Relative proportion of Tuj1+ and MyoD+ cells at different times in the nestin-GFP+ population. Notice the disappearance of MyoD expression at day 5 and the progressive increase in Tuj1+ cells to become almost the only population by days 7 and 14 in culture.

Figure 1A shows the change in appearance over time of neural phenotype cells expressing nestin-GFP and Tuj1 in FDB fiber culture. Tuj1 is commonly used as an axonal marker in young neurons [22], [23], [24]. Nestin-GFP+/Tuj1+ cells increased from day 1 to 14, representing a fraction of the total number of cells in culture, based on Hoechst 33342 staining. Merged fluorescence and brightfield images indicate that at day 1, the nestin-GFP+ cells were loose in the chamber or associated with myofibers, but all were Tuj1-, while at day 4, nestin-GFP+/Tuj1+ cells with neural phenotype were apparent. At days 7 and 14, neural cells became the dominant population among nestin-GFP+ cells.

To determine how early nestin-GFP+/Tuj1+ cells appear in our cultures, we examined their phenotype and immunoreaction profile daily during the first week and at two weeks after plating. Figure 1B shows nestin-GFP, MyoD, and Tuj1 expression in cell cultures derived from FDB muscles. Nestin-GFP allowed us to identify either muscle or neural precursor cells [15], [25], while MyoD and Tuj1 revealed myogenic and neurogenic cells, respectively [26], [27]. At day 1, myofiber nuclei and activated muscle satellite cells are MyoD+ and Tuj1-, and nestin-GFP fluorescence was obvious; 10.2±1.6% of the nestin GFP+ cells were also MyoD+. The use of collagenase type-2, an enzyme that seems to destroy myofibers' basal lamina, consequently releasing most satellite cells, may account for the large number of myofibers with no satellite cells attached [28]. Merged Pax7 and nestin-GFP images confirm the presence of quiescent muscle precursors in satellite position with respect to the myofiber (see below). Brightfield images support the presence of cells expressing nestin-GFP, either attached to satellite cells or detached from the myofiber. At day 2, the population of nestin-GFP+/MyoD+ increased to 13.3±2.4% due to proliferation of released and attached satellite cells. At the same time, a population of Tuj1+ cells appeared, representing 24.5±3.3% of the nestin-GFP+ cells. Notably, all Tuj1+ cells were also nestin-GFP+, which indicates that nestin expression at this stage fails to differentiate between the two cell progenitors. MyoD+ cells do not react to Tuj1 antibody and vice versa in more than 1,000 cells from 8 FDB muscles, supporting the concept that these two markers clearly identify two distinct cell populations: one committed to muscle and the other to neural lineages.

At day 3, neural outpaced myogenic precursor cells to become the predominant or almost exclusive population in nestin-GFP+ cells. Nestin-GFP+/MyoD+ cells gradually began to decrease, representing 9.5±3.2%, while Tuj1+ cells were already 40.4±8% of all nestin-GFP+ cells. One factor contributing to this predominance is the decline of nestin-GFP expression in muscle progenitor cells with time in culture [25].

As early as day 4, almost three-fourths of nestin-GFP+ cells (73.8±4.8%) were Tuj1+, while nestin-GFP+/MyoD+ cells represented only 2.7±2.7%. This finding is consistent with previous reports of GFP fluorescence disappearing in satellite cells that are MyoD+ at a similar time point [25].

At day 5, the population of nestin+/Tuj1+ cells increased to 88.8±0.8%, while the nestin-GFP+/MyoD+ population disappeared completely, as confirmed by time-lapse experiments (see below).

At day 6, 96±0.4% of cells were nestin+/Tuj1+, while from day 7 to day 14, they represented 100% of nestin-GFP+ cells. This co-expression indicates that nestin-GFP+ fluorescence in cells cultured for 7–14 days defines a population of neural progenitor cells in the skeletal muscle of the adult mouse. These experiments were repeated three times using 4 nestin-GFP transgenic mice, 8 FDB muscles, and 40 coverslips per experiment. Four microscope fields were analyzed per coverslip (160 fields total) at 20X magnification for cell quantification.

Note that the culture medium used for these experiments contains chicken embryo extract, which is a cocktail of multiple trophic factors and mitogenic cues, and no specific neurotrophic factors were added. This medium is normally used for myogenic cultures and to support muscle cell proliferation and differentiation [29]. To assess its importance, we used cultures without embryo extract and obtained similar results (data not shown), which implies that it is not essential for commitment to neural cells.

Laminin coating on dishes made an obvious difference. For prolonged periods, nestin-GFP+ cells in culture did not adhere to uncoated dishes, indicating that they need laminin as an attachment matrix.

Fibroblasts, myogenic cells, and pericytes may contribute to the significant number of MyoD-/Tuj1- cell nuclei in our cultures. Pericytes, a population of α smooth-muscle actin+ and CD31+ cells may contribute to the pool of nestin-GFP+ fluorescent cells during days 1–6, but they do not react to Tuj1 (see below). Approximately all nestin-GFP+ cells co-expressed Tuj1 from day 7 on, so they could not be pericytes.

Nestin-GFP+ cells, expressed as a fraction of all cells, decreased during the first 4 days in culture. At days 1–4, this fraction was 19.3±2.5%, 16.5±0.5%, 9.4±1.4%, and 5.2±0.8%, respectively, which may be explained by either a relative increase in nestin-GFP- cells or decreased nestin-GFP+/MyoD+ cells, corresponding to satellite cells and/or nestin-GFP+/MyoD- cells. After day 4, the fraction of nestin-GFP+ outpaces negative cells, being 5.8±2.2%, 9.7±1.3%, 14.7±5.0%, and 54.9±19.0% at days 5–7 and 14, respectively.

### Neural nestin-GFP+ cells do not derive from muscle satellite cells

**Figure 2:**
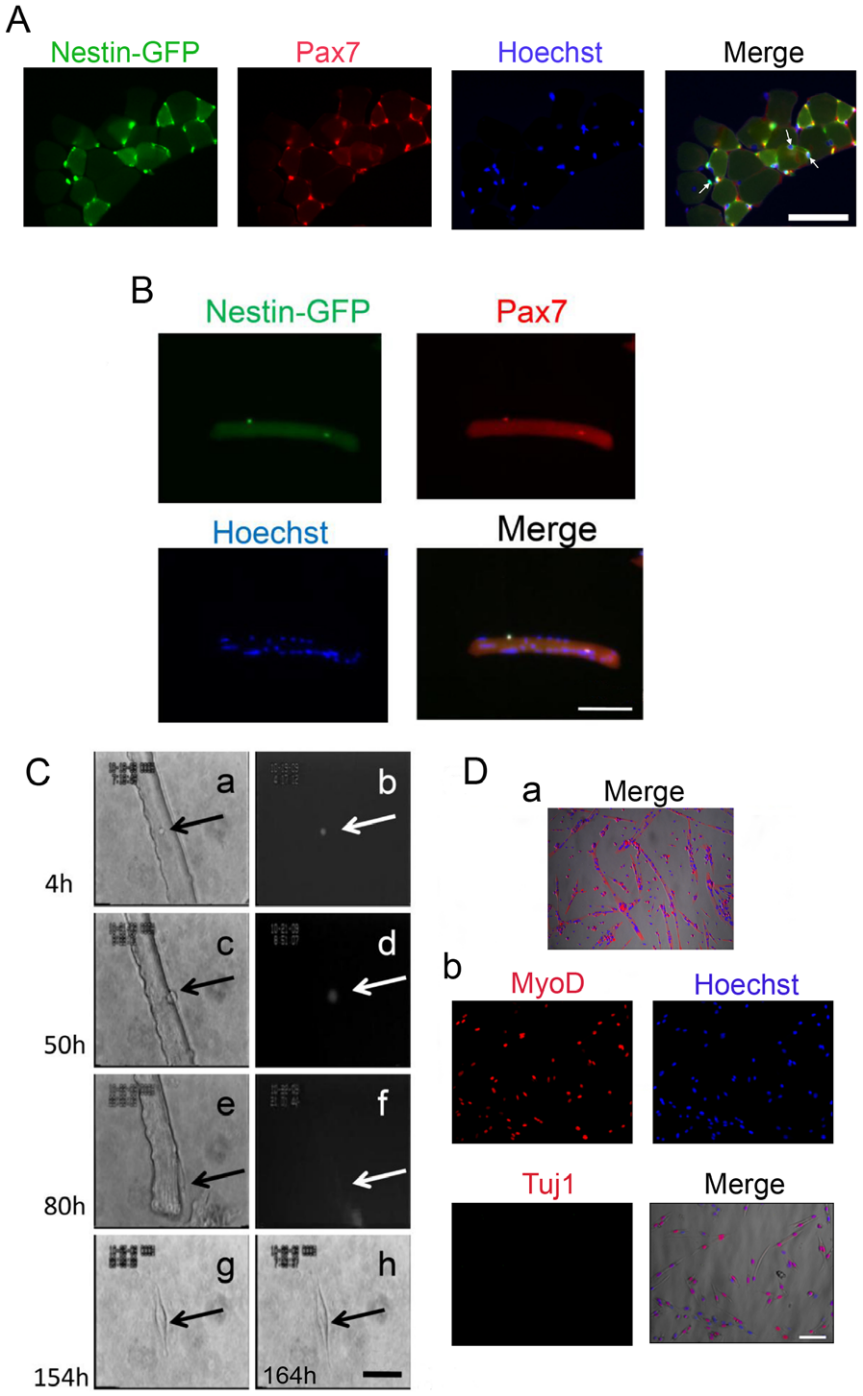
Nestin-GFP+ cells attached to myofiber (satellite cells) do not produce Tuj1+ cells with a neural phenotype. **A.** A representative EDL muscle cross-section from a nestin-GFP transgenic mouse showing GFP expression and Pax7+ immunoreaction (n = 3 mice, 6 EDL muscles). Myofibers were counterstained with Hoechst. The merge image shows examples of GFP+/Pax- cells (arrows). Scale bar  = 50 µm. **B.** Enzymatically dissociated single FDB muscle fiber showing 2 nestin-GFP+ satellite cells that immunoreact to Pax7 and overlap with Hoechst nuclear staining. Scale bar  = 100 µm. **C.** FDB satellite cell time-lapse analysis. A nestin-GFP+ satellite cell attached to an FDB fiber (arrow) analyzed for more than 6 days. Snapshots of the complete record at 4, 50, 80, 154, and 162 h. Brightfield (a, c, e, g, h) and fluorescence (b, d, f). Fluorescence completely disappeared at 162 h in culture. Scale bar  = 50 µm. **Da.** Four-month satellite cell culture from isolated EDL fibers. Myoblasts and myotubes are stained for desmin and Hoechst, which overlap with the brightfield image. **b.** The culture shown in *Da* is MyoD+ but Tuj1-. Scale bar for all pictures in D = 100 µm.

Investigating nestin-GFP+ cells in EDL muscle shows that most of them overlap with Pax7 positive cells, although a small fraction does not (arrows in Fig. 2A). We speculate that this fraction of nestin-GFP+/Pax7- cells are interstitial and gives rise to our neural cells in culture (see below,). In this preparation, we have not detected Tuj1+ cells (data not shown). Satellite cells are considered the main source of stem cells in postnatal skeletal muscle, located beneath the myofiber basal lamina [30], but there are other progenitor cells in interstitial skeletal muscle [31]. Nestin-GFP+ cells remain attached as satellite cells to the myofiber after fine dissection and enzymatic dissociation with collagenase type-1. Quiescent satellite cells are typically defined by nestin-GFP [25] and Pax-7 [32]; their immunostaining coincides with Hoechst-based nuclear localization (Fig. 2B).

We followed satellite cells attached to single FDB fibers, using a time-lapse technique that takes advantage of their nestin-GFP fluorescence. Figure 2C illustrates one of 6 individual FDB fibers in which a satellite cell in phase-contrast (a, c, e, g, h) and fluorescence (b, d, f) was analyzed 4, 50, 80, 154, and 162 h after the experiment started. These snapshots of the complete record show that at 4 h, the satellite cell is attached to the FDB fiber (a, arrow), and GFP fluorescence can be clearly discerned (b). At 50 h, the satellite cell moves to a border of the myofiber (c), and GFP fluorescence starts to fade (d). At 80 h, the myofiber shrinks, and the rounded satellite cell becomes an elongated myogenic cell (e), while nestin-GFP fluorescence is difficult to detect (f), as reported previously [25]. At 154 (g) and 162 h (h), the parent myofiber has died and moved out of the microscope's field, while the new muscle cell remains with both ends retracted (g) or, finally, elongated (h).

We also used a pure satellite cell culture to determine whether satellite cells differentiate into neural cells (Fig. 2Da). These experiments used EDL fibers because they are longer and therefore have more satellite cells than FDB. Fibers showing GFP fluorescent satellite cells and no evidence of contamination were cultured in the medium reported above for 4 months. Satellite cells formed desmin+ myoblasts and myotubes exclusively; neural-like cells were completely absent from all cultures analyzed. To further examine the presence of neural precursor cells in our purified culture, we immunostained it with Tuj1 antibody and counterstained with Hoechst. All cells in the culture were Tuj1-, and most were MyoD+ (Fig. 2Db). These data, together with time-lapse experiments, support the conclusion that myofiber satellite cells do not give rise to neural precursor cells.

### Nestin-GFP+ cells isolated from skeletal muscle can form neurospheres

**Figure 3:**
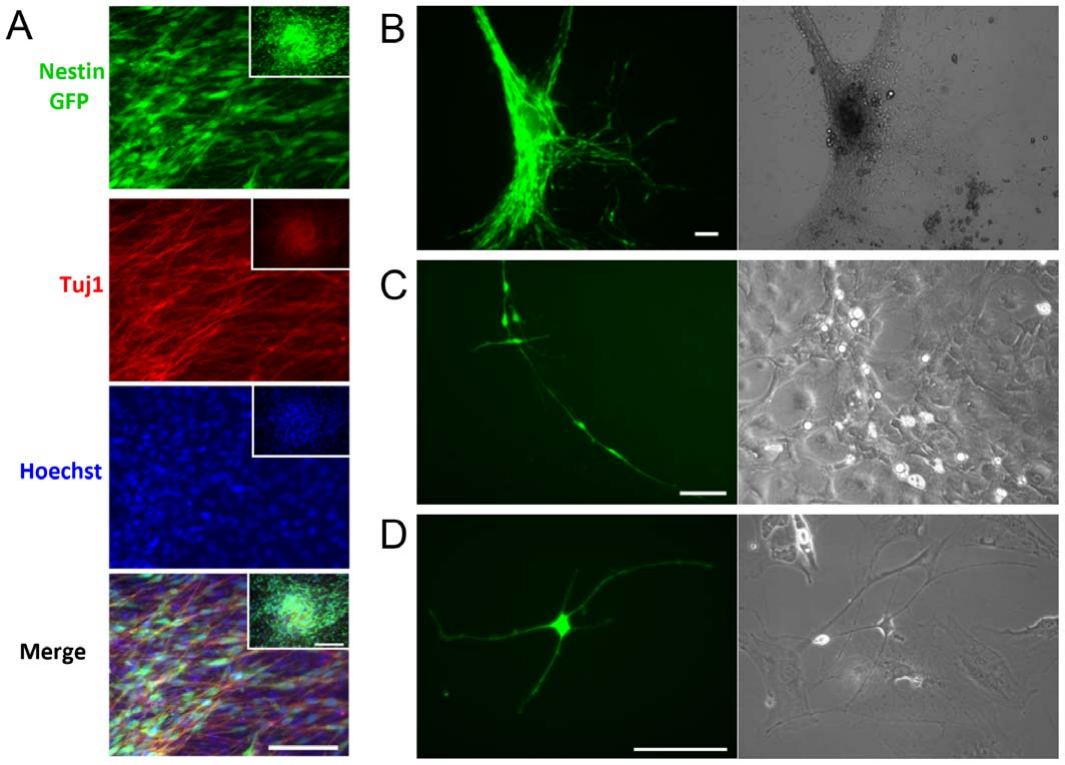
Neurospheres derived from muscle FDB cultures. **A.** Neurospheres formed on day 8 in culture are nestin-GFP+ and Tuj1+. Nuclei were stained with Hoechst 33342. **B.** Nestin-GFP+ cells grow as monolayers after fusing into neurospheres. **C.** Nestin-GFP+ cells form networks. **D.** Nestin-GFP+/Tuj1+ cells exhibit neural morphology, with many processes. Right panels for B, C, and D are brightfield images. Images in B-D were taken on culture day 8. Scale bar  = 100 µm for all pictures, including insets in panel A.

Neural progenitor cells—multipotent cells from which central nervous system neurons and glia arise—often aggregate into neurospheres, which exhibit the capacity for self-renewal [33]. Neurospheres derived from FDB muscle were characterized by morphological and immunohistochemical analysis. Nestin-GFP+/Tuj1+ cells typically formed them around day 7 in culture (n = 5 cell cultures). Most of the differentiated neurosphere-derived cells (neuron-like cells) displayed bipolar or tripolar morphology, some with 4 or 5 processes (Fig. 3A). Neurospheres spontaneously attached to the bottom of culture dishes and gave rise to cells with neural morphology. Nestin–GFP+ cells cultured from the skeletal muscle grew as monolayers in big clusters and exhibited a distinct phenotype characterized by a small cytoplasm with aligned multipolar extensions (Fig. 3B). From day 7 on, multipolar cells expressed Tuj1 protein and nestin-GFP in fairly even amounts. Brightfield images also show the predominance of a neuronal phenotype, characterized by small bright somata and two or more pronounced dendrite or axon-like processes. Incipient cell-cell contact was detected at the ends of the dendrite-like processes (Fig. 3C); however, desmosoms or fused plasmalemmas were never found [33]. Some nestin-GFP+ cells exhibited 5 or more processes (Fig. 3D). These characteristics were not evident in other cell types in the culture.

### A population of skeletal muscle nestin-GFP+ cells exhibit glutamate-evoked membrane currents

**Figure 4:**
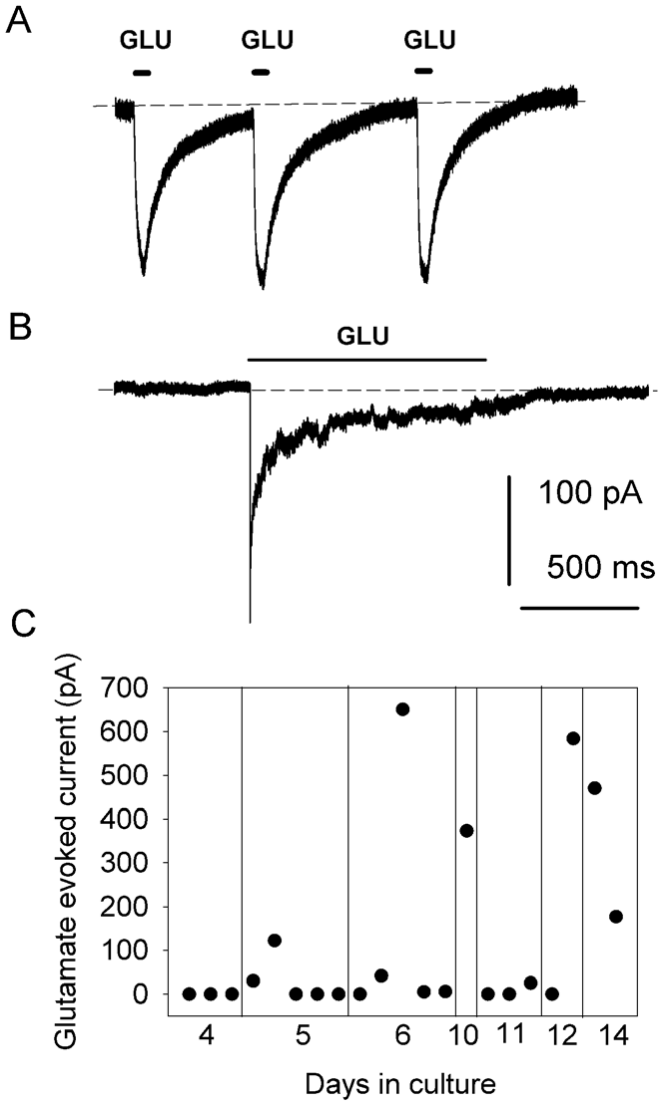
Glutamate-evoked membrane currents in neural multipolar cells under voltage-clamp. **A.** Typical inward currents in response to three 500 mM L-glutamate pulses in neural cells from FDB muscle culture. The membrane potential was held at −60 mV. Glutamate pulses are depicted above the current traces. The dashed lines represent baseline. **B.** Response to glutamate application for 1 s and desensitization during agonist application. **C.** Glutamate-evoked current as a function of culture time (days 4–14). Data points represent individual cells.

To investigate whether nestin-GFP+ cells obtained from unsorted FDB muscle culture express the ion channels typically found in neurons, we challenged them with glutamate in the whole-cell configuration of the patch-clamp. Figure 4 shows typical responses to flushing 500 mM L-glutamate over nestin-GFP+ cells with multipolar neural processes like those depicted in Figure 3D. The membrane potential was held at −60 mV, and the intracellular recording solution was devoid of Mg^2+^ (see Methods). Figure 4A shows inward currents in response to three glutamate pulses. Pulse durations are indicated above the traces. Figure 4B shows another response in a neural cell exposed to glutamate for 1 s; desensitization during the exposure is apparent. Unfortunately, these experiments did not last long enough to test currents in response to glutamate pulses at more than 2–3 membrane potentials, so channel conductance could not be calculated. An outward current at positive membrane potentials was recorded in 2 cells (data not shown). Differences in response kinetics may result from variable perfusion efficiency and/or channel expression or modulation. Figure 4C shows the 21 cells tested between days 4 and 14 in culture. Approximately 50% did not respond to glutamate pulses; 4 cells responded with a current greater than 300 pA, while the remaining 5 cells showed a current smaller than 200pA. The graph shows a small or no response to glutamate at days 4 and 5, increasing at days 12–14, which indicates the progressive differentiation and expression of ion channels in culture.

### Nestin-GFP+/Tuj1+ cells proliferate

**Figure 5:**
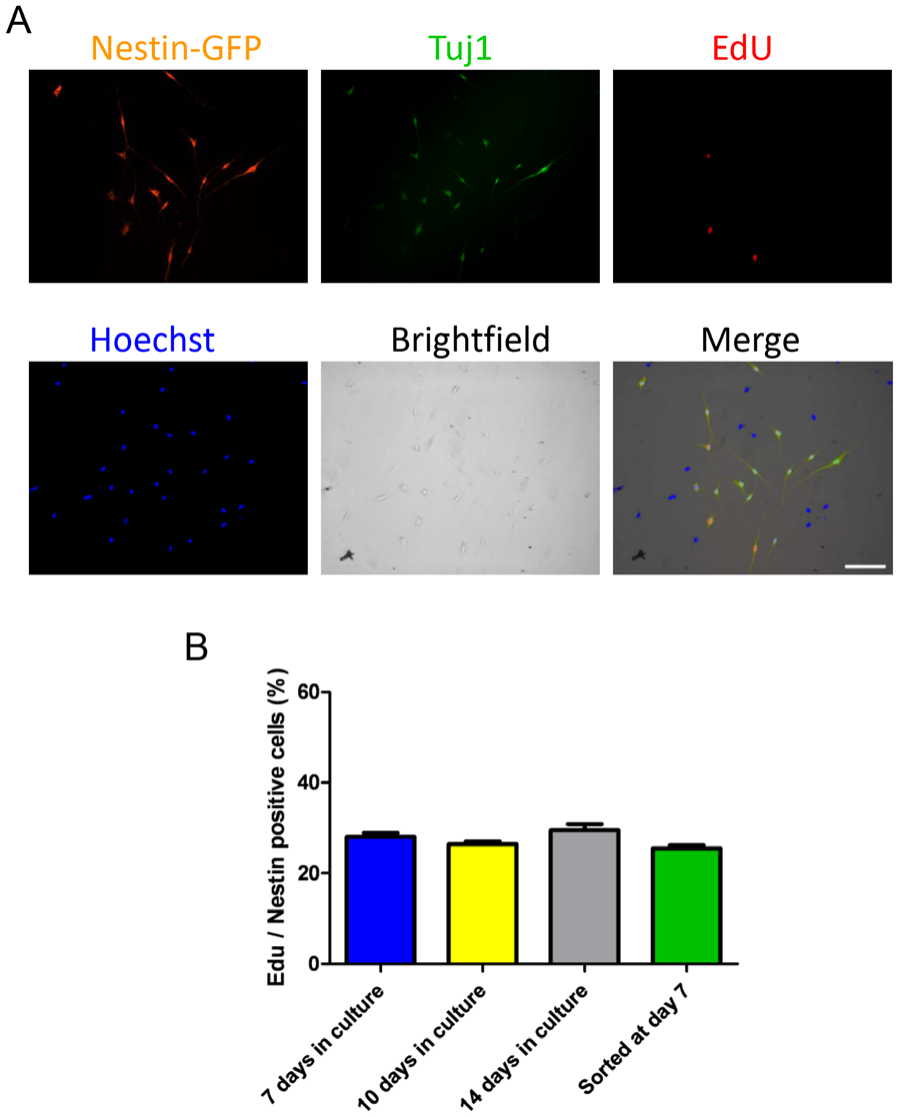
Proliferation of neural stem cells derived from FDB skeletal muscle. **A.** Unsorted nestin-GFP+ and Tuj1+ cells from FDB muscle were exposed to EdU on culture day 7. Nuclear DNA was stained with Hoechst 33342. Scale bar  = 100 µm. **B.** Percent of nestin-GFP+/Tuj1+ cells exhibiting EdU incorporation at days 7, 10, and 14 in culture. The fourth column corresponds to nestin-GFP+ cells sorted on culture day 7, cultured for 48 hours, and fixed (n = 3 preparations). EdU (10 µM) was administered 2 hours before cells were fixed. Data are mean ± SEM.

To determine whether they proliferate, nestin-GFP+ cells were cultured using the FDB preparation described above. On day 7, they were incubated with 10 µM EdU (5-ethynyl-2′ deoxyuridine) for 2 h prior to fixation and immunostaining. Results were similar to those achieved with an EdU concentration of 20 µM (data not shown), which indicates that the 10 µM EdU used in most experiments was not a limiting factor in assessing cell proliferation. Approximately 28% of nestin-GFP+/Tuj1+ were also EdU positive (Fig. 5A, B). These data support the concept that these cells proliferate. To confirm that they retain their proliferative capacity, EdU was examined at days 10 and 14: 26±0.6% and 29±1.3% cells incorporated EdU, respectively (n = 3 FDB muscles from 3 mice) (Fig. 5B). These differences were not statistically significant.

### Skeletal muscle neural progenitor cells can be purified from 7-day FDB skeletal muscle cultures based on nestin-GFP expression

**Figure 6:**
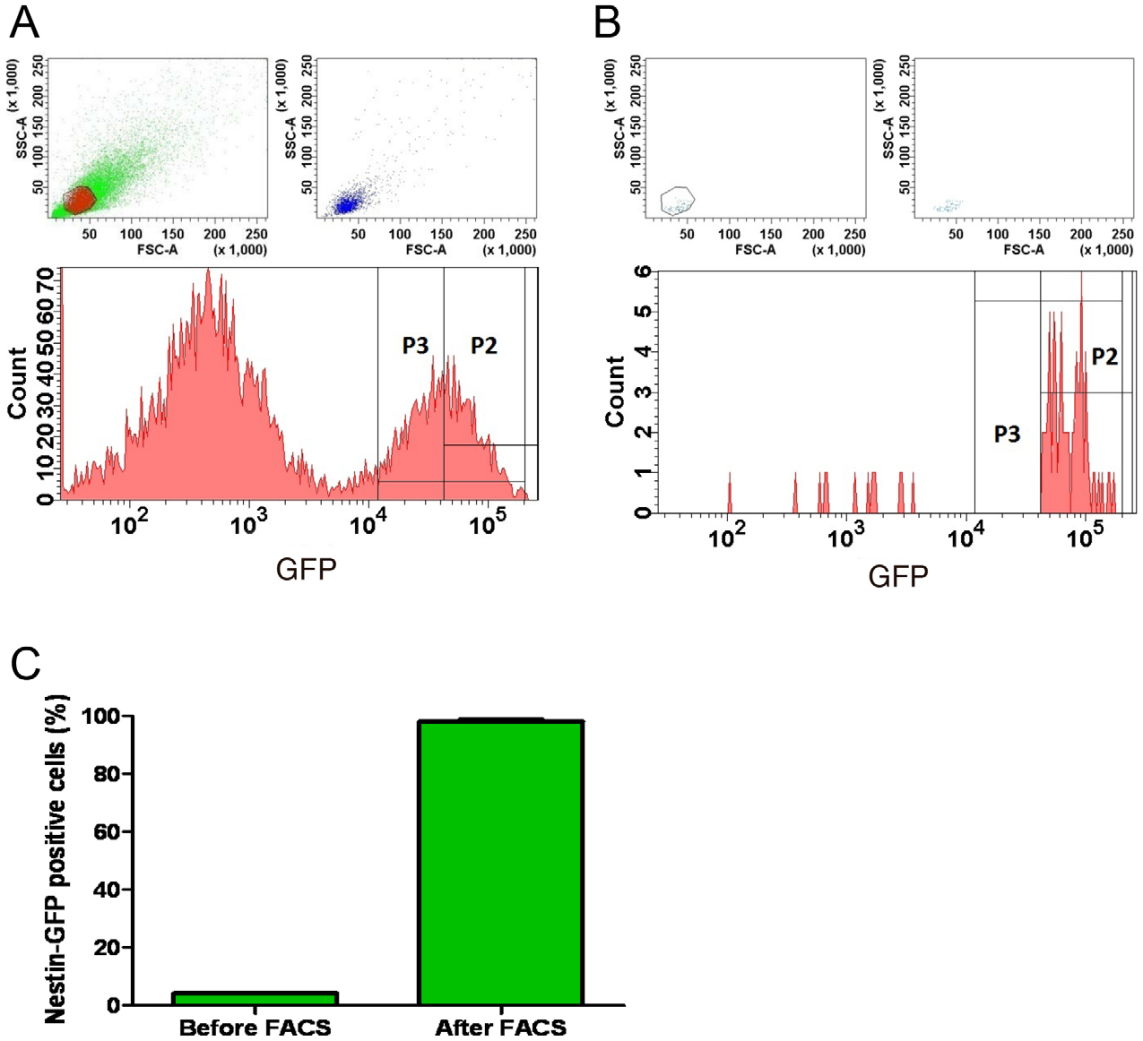
Nestin-GFP+ cell sorting. Representative scatter plots for 7-day FDB cultures, before (**A**) and after (**B**) FACS. GFP fluorescence was plotted against the number of cells. We selected cells with very high GFP fluorescence (P2). **A.** Sorting yielded approximately 5% of total GFP events and a large population of GFP- cells. **B.** Re-analysis of GFP+ cells after sorting. **C.** Percent of nestin-GFP+ cells before and after FACS. Data are mean ± SEM.

To investigate whether nestin-GFP+ cells can survive and proliferate in isolation from other cells in FDB cultures, they were sorted based on nestin-GFP fluorescence at day 7 and cultured. Flow cytometry showed that nestin-GFP+ cells represent approximately 5% of the presorted cell population (n = 3 preparations from 3 different cultures; Fig. 6A, C). We also sorted GFP- cells as a control. To verify the purity of our sorted cells, they were re-analyzed by cell sorting, which confirmed that all were GFP+ (Fig. 6B). Sorted cells were plated on dishes precoated with laminin and cultured using the medium described above (Methods).

### Sorted nestin-GFP+ cells exhibit neural markers

**Figure 7:**
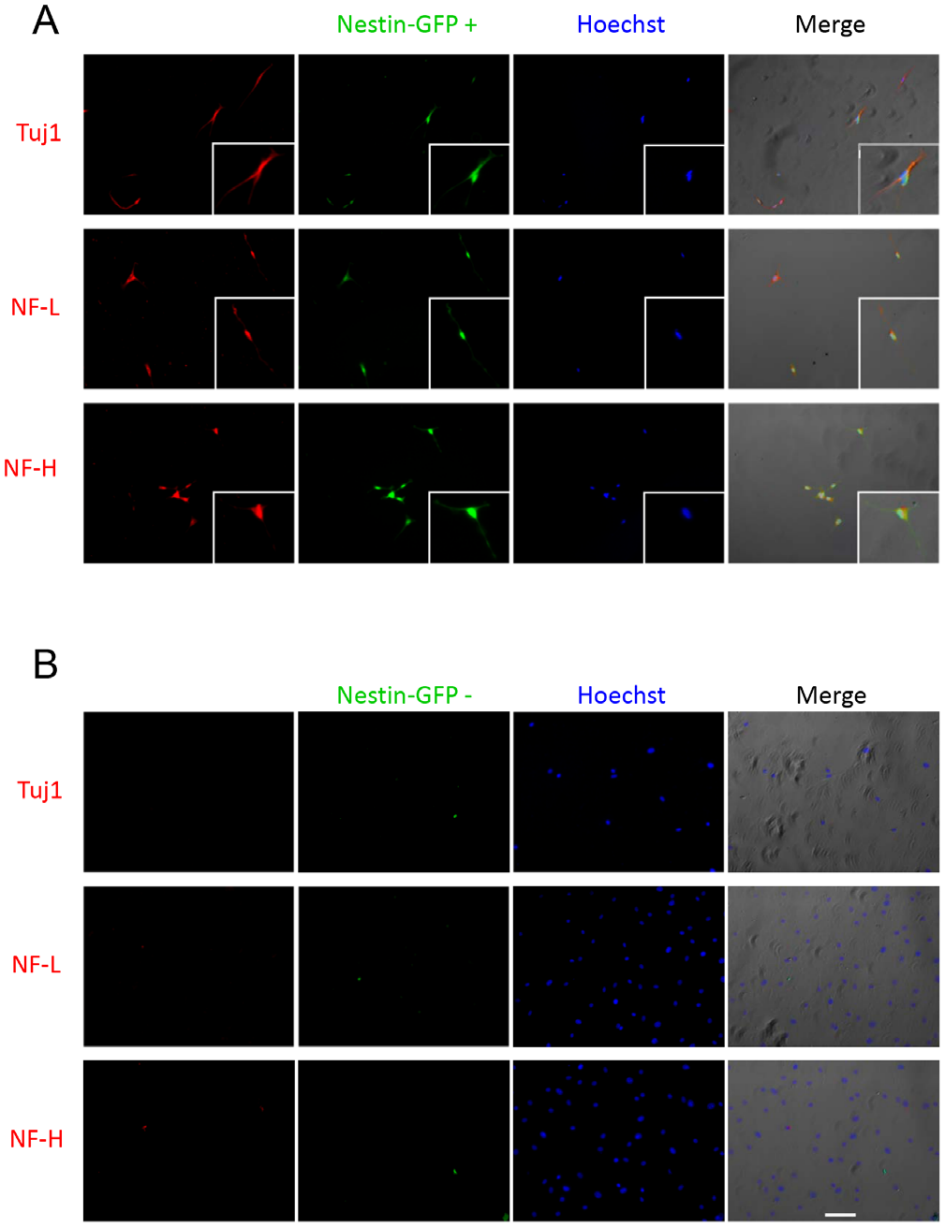
Sorted nestin-GFP+ cells from FDB cultures show neural markers. Sorted GFP+ (A) and GFP- (B) cells were seeded in parallel on dishes precoated with laminin and grown for 48 hours until attached. The first column shows immunostaining for Tuj1, NF-L, or NF-H, while the second shows their corresponding nestin-GFP fluorescence. Nuclei were stained with Hoechst. Merged images are shown on the far right. Scale bar  = 100 µm.

After 48 hours in culture, nestin-GFP+ sorted cells from the FDB muscle were examined for Tuj1, neurofilament-L (NF-L), and neurofilament-H (NF-H) expression in 4 FDB from 2 mouse cultures, which gave 4 dishes, each containing 5 coverslips; 100 cells were counted per stain. All nestin-GFP+ cells were positive for these markers (Fig. 7A). These cells survive at least 2 weeks in culture and retain proliferative capacity, as demonstrated by the number that incorporated EdU (25±0.7%) (Fig. 5B). All nestin-GFP- cells were negative for these markers (Fig. 7B). Nestin-GFP+ cell immunostaining that omitted the primary antibody was negative for the three neural markers (data not shown).

### Endothelial cell, pericyte, and Schwann cell markers in unsorted FDB muscle-derived nestin-GFP+ neural cells

**Figure 8:**
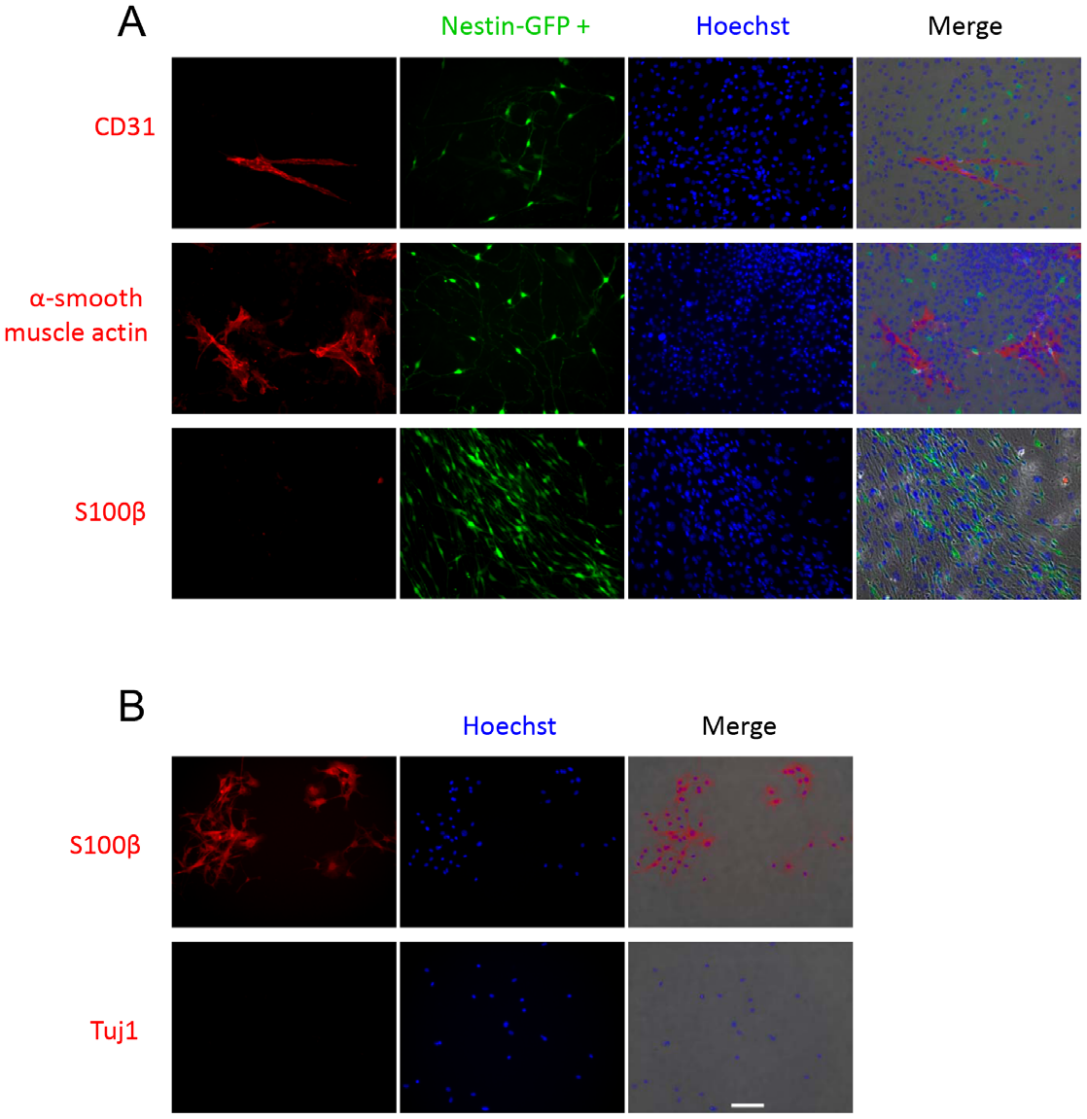
Unsorted nestin-GFP+ cells from FDB cultures do not exhibit endothelial cell, pericyte, or Schwann cell markers. Unsorted FDB-derived cells were seeded on precoated dishes with laminin and grown for 10 days. A. A CD31 antibody identified a population of endothelial cells that did not overlap with nestin-GFP+ cells. Similarly, an α smooth-muscle actin antibody recognized a population of pericytes. Nestin-GFP+ cells do not react with the S100β antibody for Schwann cells. B. A primary Schwann cell culture shows positive immunostaining when exposed to S100β but no immunostaining when incubated with Tuj1. Scale bar  = 100 µm.

We examined whether unsorted cells exhibit endothelial cell, pericyte, and Schwann cell markers in FDB muscle-derived nestin-GFP+ neural cells using CD31, α smooth-muscle actin, and S100β antibodies, respectively, at day 10 in culture. Figure 8A shows that nestin-GFP+ cells were negative to these three markers. The lack of immunoreactivity to these antibodies was confirmed in 4 FDB from 2 mouse cultures; 100 cells were counted per dish. We used a Schwann cell primary culture as a positive control for the S100β antibody to confirm that nestin-GFP+/Tuj1+ cells lack of peripheral glial marker expression. Additionally, Schwann cells did not express Tuj1 (Fig. 8B). To look for myelin production in culture, we stained cells with toluidine blue (1%) and sodium borate (1%), following published procedures [34], and observed no specific staining (n = 2 preparations, data not shown), supporting the concept that nestin-GFP+ cells are not Schwann cells or their precursors.

### Nestin-GFP+ neural stem cells derive from interstitial progenitor cells in skeletal muscle

**Figure 9:**
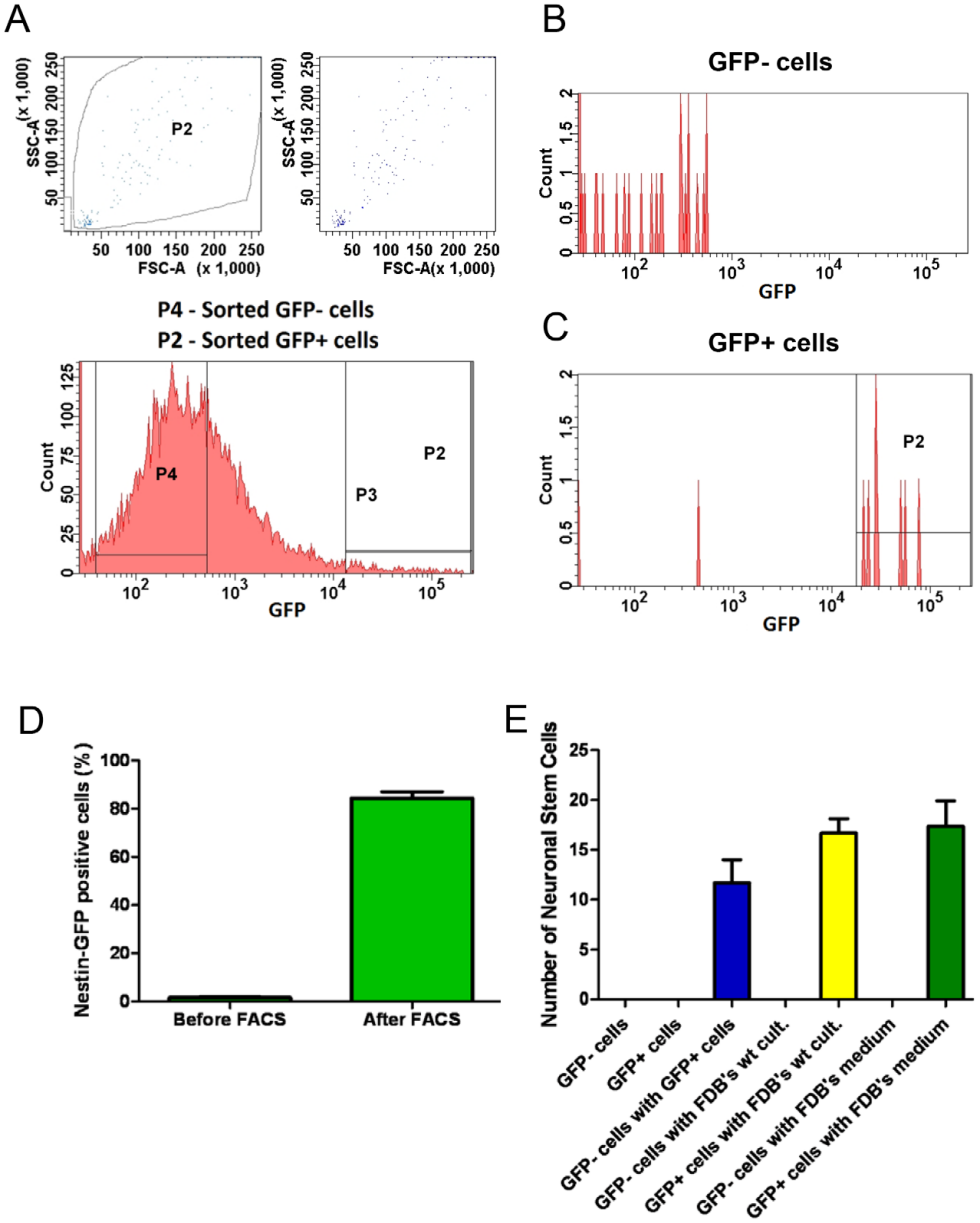
Nestin-GFP+ cell sorting and culture. **A**. Representative scatter plots showing fluorescence-activated sorting of cells derived from nestin-GFP+ hindlimb muscles immediately after isolation. GFP fluorescence was plotted against the number of cells. We selected cells with very high (P2) and no GFP fluorescence (P4). Sorts of total events yielded 1.9% GFP+. Histograms of re-analysis of sorted GFP- (**B**) and GFP+ cells (**C**). Note that B shows some GFP- cells, which probably result from a decline in fluorescence with time. **D.** Percent of nestin-GFP+ cells before and after FACS. **E.** Number of nestin-GFP+ cells differentiated into neurons after culture in different conditions: nestin-GFP- or nestin-GFP+ cells cultured alone; co-cultured nestin-GFP+ and nestin-GFP- cells; nestin-GFP- or nestin-GFP+ cells co-cultured with wild-type FDB fibers; and nestin-GFP+ or nestin-GFP- cells co-cultured with medium from wild-type mouse FDB analyzed after 8 days in culture. The number of nestin-GFP+ and nestin-GFP- plated cells were approximately 5000 and 40,000 per dish, respectively, in all conditions. Only GFP+ cells with 2 or more processes were counted. Data are mean ± SEM.

To address the source of nestin-GFP+/Tuj1+ neural stem cells, immediately after isolating hindlimb muscles, we sorted two populations, GFP+ and GFP-, and cultured them separately under different conditions (Fig. 9A–C). Nestin-GFP+ cells represent a small fraction of all sorted cells (Fig. 9D), as reported above. A comparison between nestin-GFP+ cells sorted from pooled hindlimb muscles and FDB muscles is not straightforward. Pooled hindlimb cells were sorted immediately after dissociation, normalized to the number of events, and characterized by a significant number of floating cells, while FDB muscle cells were sorted after 7 days in culture, normalized to the number of nuclei, and floating cells were absent. These methodological differences affect whether these two preparations yield variable nestin-GFP+ cells and require further examination. Neither positive nor negative cells gave rise to cells with neural phenotype after 8 days in culture (Fig. 9E), which agrees with previous observations [25]. However, when co-cultured, approximately 12 nestin-GFP+/Tuj1+ cells/dish showed 2 or more processes. To determine the source of these neural cells, either nestin-GFP+ or nestin-GFP- cells were co-cultured with dissociated FDB muscle fibers from wild-type mice. While the negative cell co-culture produced no cells with neural morphology, that with positive cells produced approximately 17 cells/dish with neural morphology, supporting the concept that nestin+/Tuj1+ cells arise from nestin-GFP+ progenitors residing in skeletal muscle. To exclude the influence of myofibers, before culture, FDB preparations were filtered through a 40 µM strainer that retained the myofibers. After 7 days in culture, cells with neuronal phenotype co-expressing nestin-GFP and Tuj1 appeared normal (data not shown). To test the action of putative factors secreted by nestin-GFP- cells on nestin-GFP+ cell morphology, the medium from the FDB cultures was added to nestin-GFP+ cultured cells. Approximately 17 cells/dish showed neural phenotype, indicating that the medium contains soluble “factor(s)” that enable nestin-GFP+ cells to adopt a neural phenotype.

## Discussion

### Nestin-GFP and Tuj1 expression markers can identify neural progenitor cells from adult skeletal muscle

In this study, starting at day 2 in culture, we isolated neural progenitor cells from adult skeletal muscle-derived cultures based on nestin-GFP and Tuj1 expression. These cells also expressed light and heavy neurofilament as additional neural properties but not CD31, α smooth-muscle actin, or S100β, which indicates that they express typical neural but not endothelial cell, pericyte, or Schwann cell markers. Here, we propose that the continuous expression of transgenic nestin-GFP in muscle cultures provides an invaluable tool to isolate a population of predifferentiated neural cells with therapeutic potential.

Neural stem cells isolated from the embryonic central nervous system express nestin, a neuro-ectodermal cell marker [14]. After a few days *in vitro*, when plated on a coated surface, they spontaneously express classical markers of more differentiated neural cells, such as Tuj1 [35], and recapitulate major features of central nervous system neural cell generation [36], [37]. Based on these observations, we conclude that cells that express nestin and Tuj1 are neural progenitors. Under our culture conditions, nestin-GFP+/Tuj1+ cells seem to produce only predifferentiated neural cells, possibly from a reduced capacity to produce other cell types [38]. Whether they might produce a more diverse cell population in animals of different species or age is not known.

Previous studies showed GFP+ neural progenitors in the brain [15] but not the skeletal muscle of the nestin-GFP transgenic mice used in this work [39], which may be explained by differences in cell isolation and culture [25]. In our work, cells sorted after 7 days in culture were already predifferentiated, while in Day's publication, nestin-GFP+ cells were sorted and cultured at an earlier time. Their lack of neurogenic capacity indicates that neural progenitor cells need the influence of nestin-GFP- cells to develop a neural phenotype.

### Nestin-GFP+/Tuj1+ neural progenitors originate from interstitial but not myofiber satellite cells

Nestin-GFP+ cells are located beneath the basal lamina in a myofiber satellite position and the interstitium of the skeletal muscle. In our culture conditions, nestin-GFP+ cells attached to myofibers are quiescent satellite cells, based on Pax7+ immunoreaction, and exhibit exclusive myogenic potential. Analysis of skeletal muscle cross-sections from the nestin-GFP transgenic mouse shows population of GFP+/Pax7- cells, which suggests that cells other than quiescent satellite cells express GFP under nestin intron II control in the native muscle. These cells may correspond to activated satellite, neural progenitor, or some other cells. We ruled out the possibility that satellite cells produce neural cells because myogenic cells lose nestin-GFP expression by days 4–5 in culture, as reported previously [25], and satellite cells released enzymatically from myofibers differentiate into a myogenic but not neurogenic lineage, based on prolonged culture (4 month), morphology, staining/immunocytochemical analysis, and time-lapse experiments. This prolonged culture stains positive for desmin and MyoD but not for Tuj1, which indicates that satellite cells do not give rise to neural precursor cells. Recently, using the same transgenic mouse used here [40], nestin was shown to identify sympathetic nervous system-innervated perivascular stromal cells, which supports the concept that muscle nestin-GFP+/Tuj1+ neural cells derive from interstitial mesenchymal stem cells.

### Advantages of our experimental approach, biological significance, and potential therapeutic contribution

Neural progenitor cells derived from adult skeletal muscle have been reported previously [7], [11], [41], [42]. These studies provided the groundwork for analysis of neurogenic cells in peripheral tissue; however, some limitations must be overcome to move ahead with their clinical application. A heterogeneous population of endothelial, vascular smooth, skeletal, and cardiac muscle cell progenitors was isolated, but after *in vitro* differentiation, neurally committed cells did not exceed 10% of the total [7]. Probably due to its multipotentiality, the skeletal muscle-derived culture was therapeutically ineffective, as demonstrated by lack of neural regeneration after cell engraftment in spinal cord lesions [7]. In contrast, our approach isolates a pure neural progenitor cell population, expressing nestin-GFP and Tuj1 from skeletal muscle cultures and shows a promising capacity to induce neural regeneration. Cells other than neural progenitors may express nestin-GFP in our skeletal muscle-derived culture, but we demonstrated that all nestin-GFP+ cells are neural progenitors from day 7 on. Pericytes are unlikely to contaminate our cultures, based on the absence of their typical phenotype, which is flat and fibroblast-like [43], and lack of overlap with nestin-GFP+ cells after one week in culture.

### Neurosphere formation, immunoreactivity to neural markers, and survival in culture

This work shows that nestin-GFP+ neural progenitor cells can expand into neurospheres. Our primary muscle-derived cell cultures showed the floating nestin-GFP+ spherical aggregates characteristic of neurospheres [44], [45]. Neurospheres attached and detached from the culture substrate, but in contrast to previous publications [33], their composition and the cells' morphology were more homogenous, supporting the similarity of their stem-like properties. These cells co-express nestin, which is a marker of neural progenitor cells [46], and Tuj1, commonly used as an axonal marker in young neurons [24], [46]. After 8 days, the neurospheres retain nestin and Tuj1 expression and continuously produce outgrowing cells.

One of the defining features of neural progenitors and stem cells in general is their ability to sustain multiple rounds of amplification. Nestin-GFP+/Tuj1+ cells derived from skeletal muscle cultures proliferated at a rate approximately one-fourth that of cell propagation. Moreover, these cells survive for at least two weeks and maintain their proliferation rate even when cultured after sorting. With EdU, we detected a skeletal muscle neural progenitor cell proliferation rate similar to that reported for neural progenitor cells in the hippocampus [47] and spinal cord [48].

### Functional response to glutamate

Functional analyses of our predifferentiated neural progenitor cells revealed that a population of nestin-GFP+ cells isolated from skeletal muscle had the electrophysiological characteristics of neurons. Glutamate-evoked currents shared some properties with those reported in native mouse central neurons [21]: they were evoked in a 1 mM Ca^2+^ solution; the current was inward at negative potentials; it responded to repeated glutamate challenges in a typical way; and it desensitized during prolonged glutamate application. Additionally, the number of cells responding to the agonist increased with time in culture. These recordings agree with a previous report in which intracellular Ca^2+^ concentration increased in response to glutamate [42]. The increased responsiveness of nestin-GFP+/Tuj1+ cells in culture supports their progressive differentiation and expression of ion channels.

### Nestin-GFP+/Tuj1+ cells exhibit additional neural markers and proliferation

Nestin is expressed in both embryonic and adult CNS stem cells. In embryonic CNS, its expression is closely associated in space and time with the proliferation of neuronal progenitor cells [14]. It is also expressed in adult stem cells both *in vivo* and *in vitro* under nondifferentiating conditions [49]. To confirm that our cells are neural precursors, other markers were tested. After 7 days in culture, these cells expressed Neu-H and Neu-L, evidencing predifferentiation at later stages, but they still expressed nestin-GFP, a stem cell marker. As neurons differentiate from their precursors, they downregulate nestin expression, which is replaced by other tissue-specific intermediate filaments, such as neurofilaments [14]. The fact that nestin-GFP fluorescence does not disappear after two weeks in culture indicates that neural cells have not completely matured. That nestin expression can be re-induced after it disappears in various degenerative and regenerative conditions in the fully differentiated organism has been reported [50]. A population of cells in the brain cortex co-expresses bromodeoxyuridine in Tuj1+ and Neu-L cells, suggesting generation of neurons with proliferative capacity [51]–[52]. Tuj1 is initially expressed in immature neurons and persists as they mature [23].

### Likelihood of success in transplanting predifferentiated neural progenitor cells

Our method can efficiently isolate immature neural progenitor cells from skeletal muscle culture. Predifferentiated neural precursor cells, cultured after sorting, may have a better chance for success in proliferating and replacing damaged tissue than more immature cluster cells or neurospheres. Restricting progenitor cell lineage may limit tumor formation, with better response to the tissue environment and capacity to integrate with the host tissue [53]. Several studies have indicated a higher rate of neurogenesis when a neuronal phenotype was already induced before cells were implanted [54]. Neural progenitor cells transplanted into the injured rat spinal cord favored differentiation into astrocytes but not neurons [55]. Late-stage precursors and immature neurons have been transplanted into the adult neocortex and induced pyramidal neurons to degenerate; the engrafted cells were found to follow pyramidal cell differentiation, as they were already committed to neuronal lineage [56]. Taken together, these results indicate the need for differentiation protocols before grafting.

Our results imply that, to certain extent, skeletal muscle-derived neural stem cells expressing nestin are highly proliferating and can be used for remodeling and repair processes [57], [58]. Because they can be isolated based on their nestin-GFP fluorescence, proliferation capacity, and differentiation into neurons, they can be used as a source of neural progenitor cells for therapeutic purposes in animal models of neural injury or degeneration.

### Nestin-GFP- cells promote appearance of the neural phenotype

Neural phenotype differentiation depends on some factors secreted by co-cultured cells, such as myoblasts and fibroblasts. We observed that not only nestin-GFP- cells, but also their medium, induce a neural phenotype, indicating that factors important for neuronal progenitor differentiation may be soluble, diffusible, and secreted by neighboring cells. These results are comparable to those published for co-cultures of neuronal progenitors and conditioned medium from bone marrow stromal cells (BMSCs) [59]. Fibroblasts, myoblasts, or any other cell type derived from muscle interstitium may be the source of the molecules that influence muscle interstitial cells to evolve into a neural lineage.

The main group of molecules regulating cell differentiation and proliferation are growth factors. Members of the transforming growth factor-β (TGFβ) family may influence premature neuronal differentiation [60]. Fibroblast growth factors (FGFs) are critical for midbrain/hindbrain [61] and forebrain [62] cell survival. A number of vasculature-related growth factors have been shown to regulate neural stem cell and progenitor proliferation, most notably PEDF [63] and vascular endothelial growth factor [64]. Factors secreted by blood vessels, such as the brain-derived neurogenic factor (BDNF), are known to influence neuronal stem cell behavior [65]–[66]. BDNF can also be secreted by myofibers, which play an active role as an endocrine organ [67]. Many of these factors may originate from any of the nestin-GFP- cells in our skeletal muscle-derived cultures. Additionally, strong differentiation was only observed after cells adhered to laminin-coated dishes, indicating that they require a matrix anchorage. Although necessary, laminin was not sufficient to induce a neural phenotype, as nestin-GFP+ did not give rise to neural progenitor cells when cultured in isolation.

In summary, prolonged nestin-GFP+ expression in skeletal muscle-derived cultures provides a unique tool to obtain predifferentiated neural progenitor cells. Whether these cells differentiate into multiple phenotypes or an exclusive neuron subclass is unknown. Whether they are suitable for transplantation, retain the capacity to engraft, develop specific neuronal functions, and allow tissue repair are subjects of ongoing research in our lab. The fact that an individual can be the donor and host of these cells circumvents the immunological and ethical concerns impeding cell therapy.
